# Preparation of NiAl-AlMg6 Functionally Graded Composite Using the Energy of a Highly Exothermic Ti-C Mixture during Self-Propagating High-Temperature Synthesis

**DOI:** 10.3390/ma16247584

**Published:** 2023-12-10

**Authors:** Igor Denisov, Stepan Seropyan, Andrey Malakhov, Denis Shakhray

**Affiliations:** 1 Merzhanov Institute of Structural Macrokinetics and Materials Science (ISMAN), Russian Academy of Sciences, 142432 Chernogolovka, Russia; denisov@ism.ac.ru (I.D.); malakhov@ism.ac.ru (A.M.); 2 Federal Research Center of Problems of Chemical Physics and Medicinal Chemistry of Russian Academy of Sciences, 142432 Chernogolovka, Russia; shakhray@icp.ac.ru

**Keywords:** AlMg6, NiAl, SHS, metal-intermetallic layered composite, lotus-type pores

## Abstract

A functionally graded composite NiAl-AlMg6 was prepared using the pressure of gaseous reaction products (impurity gases) produced during the synthesis of reactive powders in a sealed reactor. It has been shown that this method can be used to prepare a NiAl/AlMg6 composite with both chaotically oriented pores in the NiAl layer and unidirectionally oriented pores (lotus-type pores). The pore shape in NiAl was found to be dependent on the pressure of the impurity gases and hydrogen present in the starting titanium powder. A mechanism for pore formation in NiAl and AlMg6 composite during SHS is proposed. Thus, functionally graded high-temperature composites can be produced by SHS in a sealed reactor using the chemical reaction energy and the pressure of impurity gases and hydrogen. Additionally, minimizing the influence of impurity gases on the contact zone increases the interface area between NiAl and AlMg6.

## 1. Introduction

Composite materials with a NiAl-based intermetallic layer are promising for automotive and aerospace industries due to their high corrosion resistance, low density, high melting point, etc. [1,2,3,4]. The NiAl layer can have both a solid and porous structure, depending on the production method. Porous intermetallic layers have attracted more interest due to their unique properties, such as low density, high gas and liquid permeability, and high sound absorption [5,6,7].

NiAl can be produced by several methods, such as casting, mechanical alloying [8], spark plasma sintering (SPS) [3,9], dynamic powder compaction [10], and powder metallurgy [11]. Most of the above methods require complex and expensive equipment and are time-consuming. Combustion synthesis (CS), related to powder metallurgy, is a fast and energy-efficient method for producing NiAl and does not require special equipment. CS can be performed in two modes: self-propagating high-temperature synthesis (SHS) [12] and volume combustion synthesis [13]. SHS is preferable due to its higher energy, reaction rate, purity of the reaction products, etc. [14]. It is known that the solid-phase combustion of gasless SHS mixtures is accompanied by gas evolution due to the degassing of impurities and adsorbed gases in the starting components. Degassing has a significant influence on combustion parameters, structure, and properties of the final product [15,16]. Thus, the SHS method can be used to produce porous NiAl.

The structure and shape of the pores affect the characteristics of NiAl [13]. Multidirectional porosity in NiAl leads to disadvantages such as low viscosity and ductility at room temperature, as well as low strength and creep resistance at high temperatures [17]. These disadvantages are corrected by reducing the grain size of NiAl and creating defects in the crystal lattice by mechanical treatment [18,19]. The mechanical properties can also be improved by introducing WC, TiC, or TiB_2_ into NiAl [20,21].

The porosity of NiAl can be unidirectional. This type of porosity is commonly referred to as a lotus-type porosity, where the pores penetrate through the entire thickness of the material [22]. In Refs. [23,24], NiAl and Ni_3_Al intermetallic compounds with lotus-type porosity were fabricated by unidirectional solidification in a pressurized hydrogen atmosphere. The lotus-type porosity provides high strength compared to other porous materials. Nakajima et al. [25] provided methods for producing lotus-type porous metallic materials that possess features like elevated temperature strength and resistance to oxidation.

Thus, NiAl lotus-type porous intermetallic material is promising as a high-temperature functional material. However, using this method is challenging because it requires special equipment and pressurized atmospheres.

With all these positive properties, NiAl is preferred as an element of composite due to limited strength and ductility. Composites that combine the properties of intermetallic compounds and metals (MILs) are currently in great demand in different industries. MILs are fabricated by different methods, such as diffusion bonding, explosive welding, accumulative roll bonding, hot rolling, spark plasma sintering [26]. The major difficulty is the poor adhesion between the metal and the intermetallic layer due to the differences in properties.

The aim of this work was to prepare NiAl with multidirectional and unidirectional porosity by the SHS method and to prepare a functionally graded composite NiAl-AlMg6 by using the pressure of gaseous reaction products (impurity gases) produced during the synthesis of the starting powders in a sealed reactor. The morphology and elemental distribution along the interface and in the NiAl layer were studied through scanning electron microscope (SEM) and energy-dispersive spectroscopy (EDS). Confocal laser scanning microscopy was used to determine the geometric sizes of the pores.

## 2. Materials and Methods

To obtain composite material NiAl+AlMg6 with gradient structure, the SHS process in a sealed reactor was used. SHS is a technological process for obtaining materials based on exothermic chemical reaction of interaction of reactants in the form of combustion. Here, combustion is not the usual reaction of oxidation of powdery substances with oxygen to form the corresponding gaseous oxides but a strongly exothermic reaction of interaction of powdery reagents with each other.

**Figure 1:**
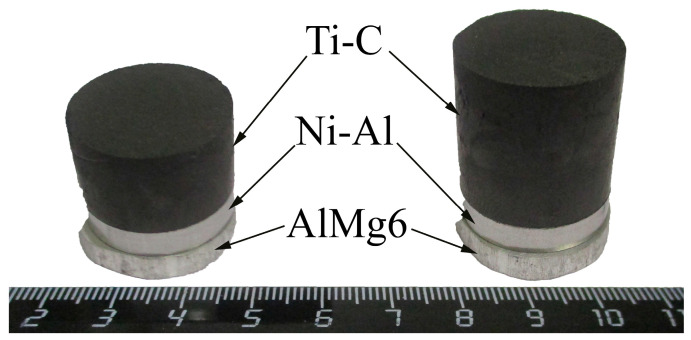
Photograph of the starting materials.

In the experiments, an AlMg6 alloy disk with a diameter of 20 mm and a thickness of 4 mm was used as a substrate. The top surface of the substrate was cleaned from oxide film using SiC grinding paper. Compressed pellets of Ni–Al and Ti-C powder mixtures were placed on the substrate (Figure 1). The thickness of the Ni–Al pellets was 4 mm and the thicknesses of the Ti-C pellets were 10 mm and 20 mm, respectively. Commercial Al powder (99.2% Al, ASD-1, Valkom-PM, Volgograd, Russia), Ni powder (99.9% Ni, PNK-UT-3, Metsintez, Moscow, Russia), Ti powder (PTS-1), and C powder (P-803) were used as precursors, with stoichiometric proportions corresponding to NiAl and TiC. To fabricate the Ni–Al/Ti-C powder mixtures, the precursors were mixed in a tumbling drum mixer for 3 h at 30 rpm with a ball-to-powder weight ratio of 5:1. The green powder mixtures were pressed into Ni–Al pellets on a manual hydraulic press (PRG-10, Lab Tools, St. Petersburg, Russia) for about 1 min at a pressure of approximately 240–250 MPa. The density of the pressed Ni–Al pellets was established through measurements of their geometric dimensions (micrometer by Dasqua, Cornegliano Laudense, Italy) and mass (CAS XE-300 analytical balance, CAS, East Rutherford, NJ, USA). The relative density was 0.75 for Ti-C pellets and 0.65 for Ni–Al pellets.

**Figure 2:**
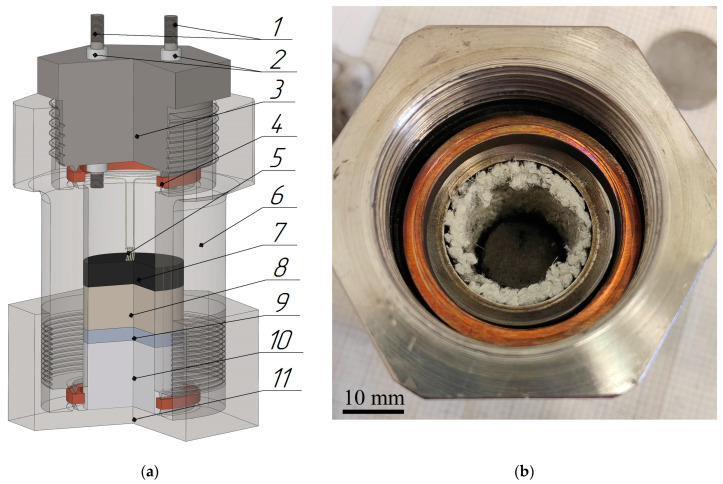
SHS reactor: (**a**) schematic diagram of the SHS reactor 1—current source, 2—insulators, 3—screwed top, 4—tightening rings, 5—electric spiral, 6—holder, 7—Ti-C pellet, 8—Ni–Al pellet, 9—disk of AlMg6, 10—heat-insulating material, 11—screwed bottom; (**b**) photograph of the SHS reactor (top view).

The Ti-C pellet was used to generate an external pressure produced during the reaction due to the release of impurity gases. The exothermic reaction in the Ti-C pellet was induced by heating with a tungsten spiral. Experiments were conducted in a special air-tight reactor (Figure 2a). The space between the reactor walls and the samples was filled with a heat-insulating layer to minimize heat loss during SHS (Figure 2b). Metallographic samples were prepared on a metallographic grinding and polishing machine (ShLIF-1M/V, TMK Engineering, Bryansk, Russia) using diamond paste. The microstructure study and energy dispersive analysis was performed on a Carl Zeiss LEO SUPRA 25 ultrahigh resolution field auto emission scanning electron microscope (Carl Zeiss AG, Oberkochen, Germany) and a Zeiss Axio Imager A1 (Carl Zeiss AG, Oberkochen, Germany) microscope. In this study, microhardness (HV) was measured using a PMT-3 Vickers hardness tester. Loads of 100 g were applied for 15 s. A confocal laser scanning microscope (Optelics Hybrid LaserTec, Yokohama, Japan) was used to obtain confocal images and 3D visualization of the surface of the samples, as well as to determine the geometric sizes of the pores.

## 3. Results and Discussion

**Figure 3:**
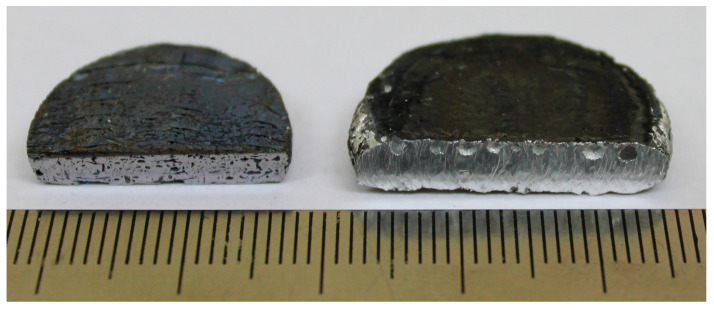
Photograph of the NiAl sample (**left**) and the AlMg6 (**right**) substrate after SHS.

When the thickness of the Ti-C pellet was 10 mm, NiAl did not interact with the AlMg6 substrate (Figure 3). However, pores with a diameter of 1.0 to 1.5 mm were uniformly distributed along the entire surface of the AlMg6 substrate.

Impurity gases produced during the Ni–Al combustion prevent interaction between NiAl and the AlMg6 substrate. This leads to the breaking of contact and adhesion between NiAl and AlMg6. It should be noted that the surface of the AlMg6 substrate, which was in contact with NiAl, partially melted. Pores in the AlMg6 substrate indicate the dissolution of an appreciable amount of adsorbed hydrogen (2–3 wt.%) contained in the powder (PTS-1) in the molten part of AlMg6. Hydrogen is considered to be the major cause of pore formation during the crystallization of aluminum alloys [27].

The mechanism of pore formation in AlMg6 is as follows: Hydrogen from PTS-1 accumulates in a thin layer of liquid metal next to the combustion front and transfers to the AlMg6 layer during crystallization. Then, pores are formed during melt cooling, when atomic hydrogen accumulates in the liquid metal layer due to a sharp drop in solubility and combines with crystallization centers and impurities. Finally, the liquid metal solidifies quickly, trapping hydrogen-filled spherical voids on the surface of AlMg6 and forming pores. It is worth noting that Denisov I. et al. [28] did not find such pores when SHS was conducted in a cylindrical powder pellet holder. This is due to the fact that the pellets in [28] were smaller, and the cylindrical powder pellet holder was not sealed enough to create conditions for the formation of pores in the AlMg6 layer.

**Figure 4:**
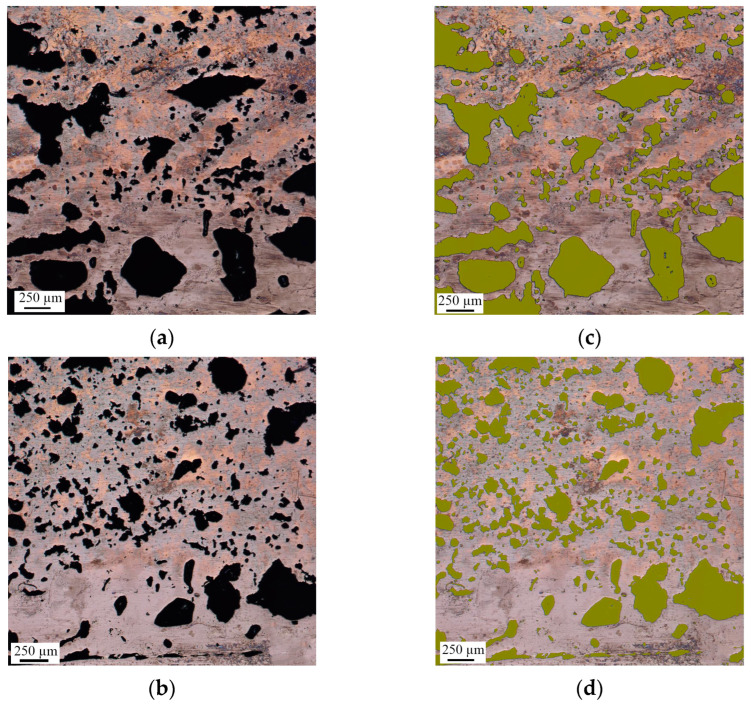
Microstructure of the upper part of the NiAl sample: (**a**) optical micrograph of the peripheral part; (**b**) optical micrograph of the central part; (**c**) confocal image of the peripheral part; (**d**) confocal image of the central part.

The microstructure of the NiAl intermetallic compound is porous with closed, elongated, small (≤50 µm), and large (≥50 µm) pores (Figure 4a,b) distributed in a chaotic manner. The surface area occupied by pores in the NiAl sample closer to the lateral periphery is about 28% (Figure 4c) and about 24% in the central part (Figure 4d). This pore distribution is explained by the release of impurity gas in the central part of the pellet during SHS. This gas migrates to the periphery and meets other impurity gases, resulting in the formation of larger pores.

**Figure 5:**
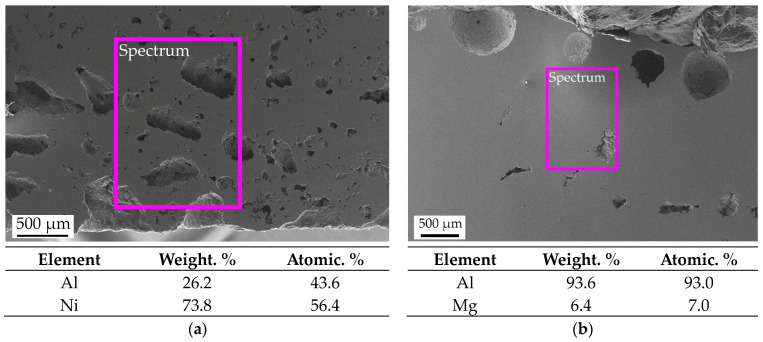
SEM image and EDS results: (**a**) NiAl; (**b**) AlMg6. the scanning area is indicated by a purple rectangle.

SEM images and EDS analysis of the micro-sections of synthesized NiAl and the surface of the AlMg6 substrate are shown in Figure 5. EDS analysis showed that during the synthesis of NiAl, the diffusion of Al and Mg between NiAl and AlMg6 does not occur.

**Figure 6:**
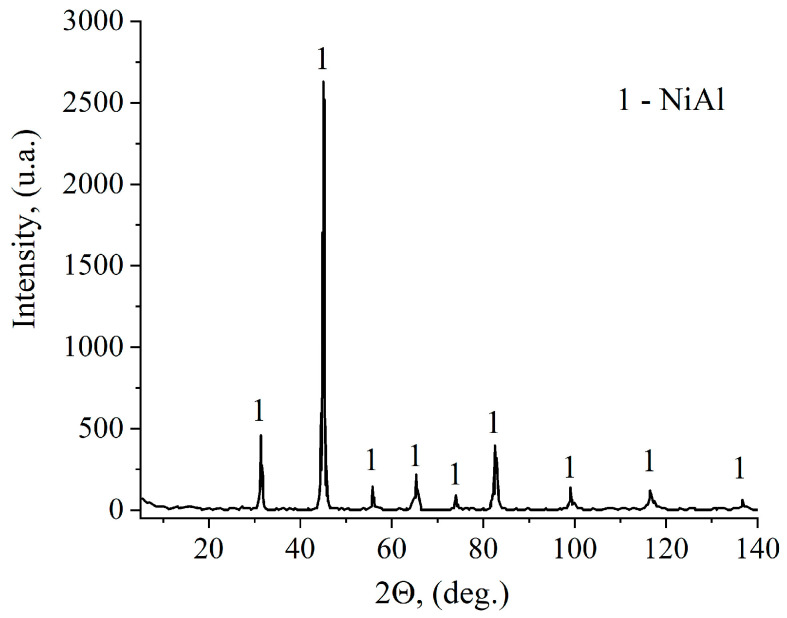
XRD pattern of NiAl (Ti-C pellet is 10 mm in thickness).

The X-ray diffraction showed that the synthesis of the Ni–Al mixture resulted in monophasic NiAl (Figure 6). 

**Figure 7:**
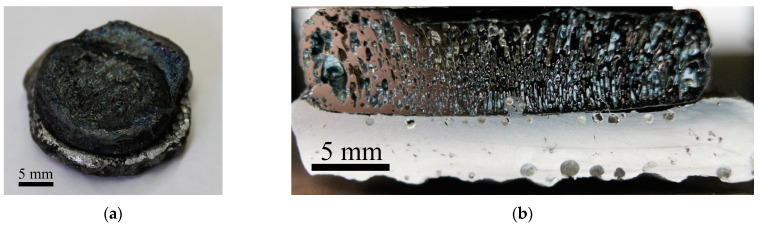
NiAl-AlMg6 sample after SHS: (**a**)—photograph of the sample; (**b**)—photograph of the sample cross-section.

A two-layer material was synthesized using a 20 mm thick Ti-C pellet (Figure 7a). However, the interface area between NiAl and AlMg6 was small. Only localized zones of layer adhesion were detected (Figure 7b). Thus, the pressure achieved during the SHS process in the reactor partially compensated for the pressure produced at the interface between NiAl and AlMg6, resulting in the formation of diffusion centers between the layers.

In addition, the high pressure and temperature in the reactor deformed the rear surface of AlMg6, which was in contact with the heat-insulating material.

**Figure 8:**
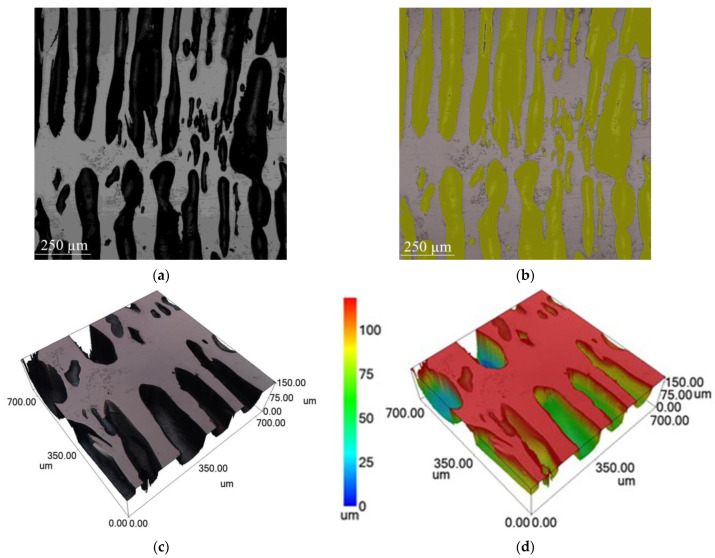
Microstructure of lotus-type pores in NiAl: (**a**) optical micrograph of lotus-type pores in NiAl; (**b**) confocal image of lotus-type pores in NiAl; (**c**,**d**) confocal 3D image showing geometric dimensions of lotus-type pores.

The NiAl layer had a porous structure with cylindrical pores (Figure 8). Pores of similar shape were observed by Ide et al. [24], and the authors called them lotus-type pores.

In our case, lotus-type pores form when impurity gases pass through solidifying NiAl under pressure from gaseous reaction products in a closed space. The lotus-type pores are vertical (Figure 8a,c) in the center of the sample but become curved closer to the periphery. The surface area of the pores is 53% (Figure 8b), and the pore diameter ranges from 70 to 130 µm (Figure 8d).

**Figure 9:**
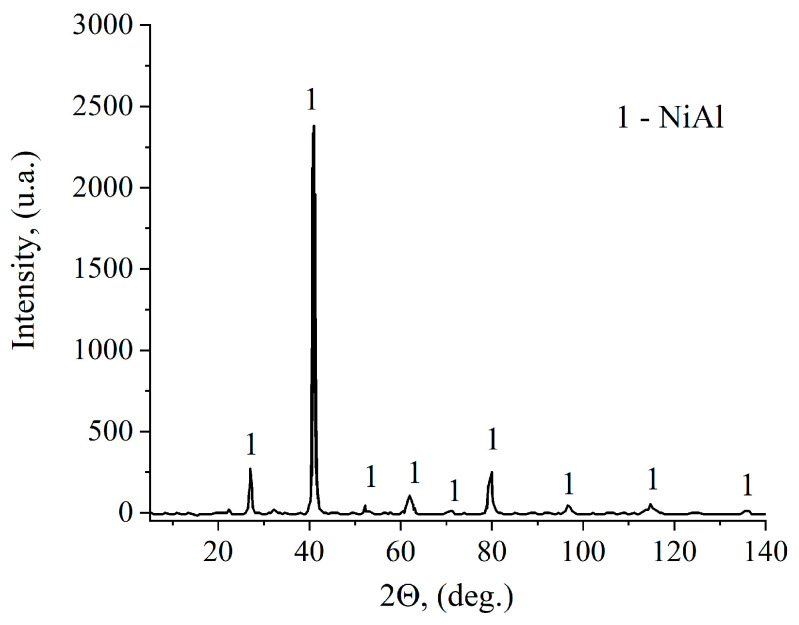
XRD pattern of NiAl (Ti-C pellet is 20 mm in thickness).

X-ray diffraction showed that the synthesis of the Ni–Al mixture produced monophasic NiAl (Figure 9).

**Figure 10:**
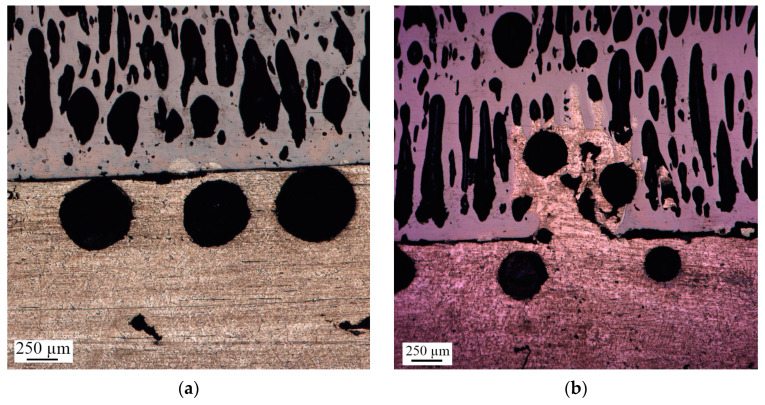
Microstructure of the NiAl-AlMg6 interface: (**a**) section with interface delamination; (**b**) section with lotus-type pores filled with AlMg6 alloy.

Detailed studies revealed a rectilinear interface between NiAl and AlMg6, with delamination regions throughout (Figure 10a). Spherical pores of the same type as in the first experiment were found near the interface of AlMg6 layers. Lotus-type pores filled with AlMg6 elements were observed at the layer interfaces to a depth of 300 µm (Figure 10b). Apparently, the molten AlMg6 layer penetrates these pores at the bottom of the NiAl pellet by capillary forces, forming an interface.

**Figure 11:**
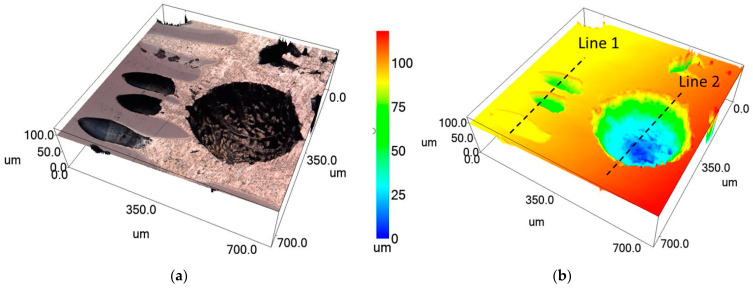

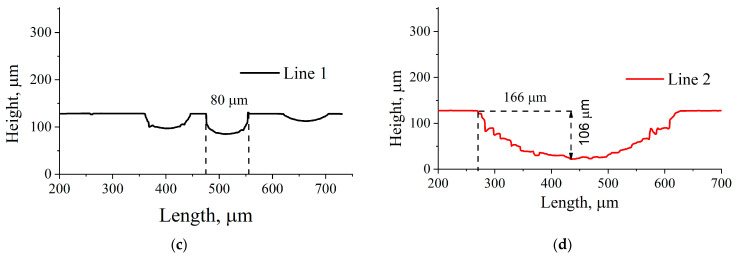
Microstructure of the NiAl-AlMg6 interface (**a**) confocal 3D image; (**b**) geometric dimensions of lotus-type pores; (**c**) geometric dimensions of pores in NiAl; (**d**) geometric pore size in AlMg6.

The study of the geometric parameters of the pores showed that the pores have a diameter of 70 to 80 µm (Figure 11a–c) and are distributed over the entire thickness of the NiAl intermetallic compound. The pores in AlMg6 have a diameter of about 300 to 340 µm and a depth of 100 to 110 µm. Thus, the pores in AlMg6 at the interface with NiAl are shaped like flattened spheroids with a volume of 0.011–0.012 mm^3^.

**Figure 12:**
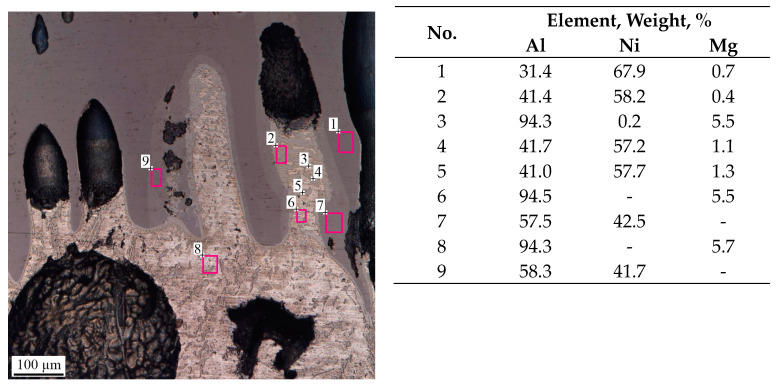
SEM image of the NiAl/AlMg6 interface and the results of EDS analysis. The results of EDS area (№1, 2, 6–9) and point analysis (№3, 4, 5).

SEM images show that the diffusion interaction between AlMg6 and NiAl in the pores (Figure 12) resulted in the formation of a Ni_2_Al_3_ phase at the NiAl/AlMg6 interface and a NiAl_3_ phase in AlMg6 over the entire diameter. Diffusion interaction was possible due to the lower heat dissipation inside the pores compared to the heat dissipation at the NiAl/AlMg6 interface, which resulted in a longer contact of NiAl with the AlMg6 melt.

**Figure 13:**
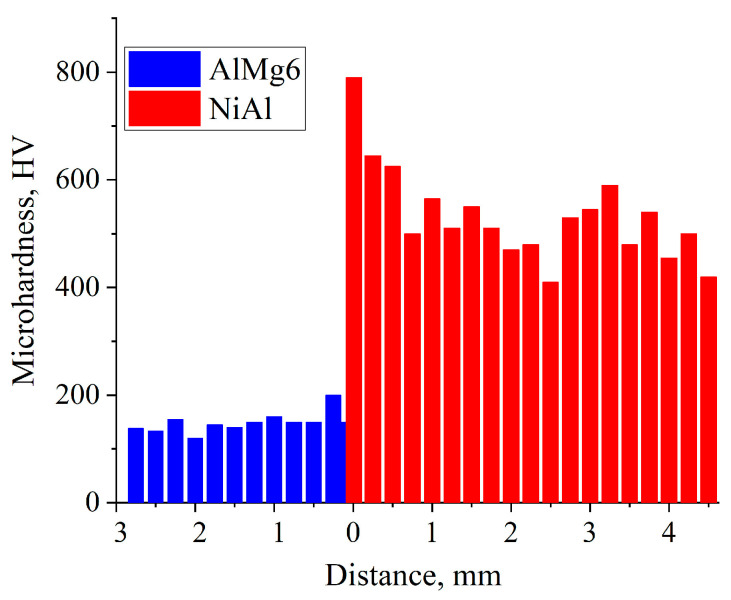
Microhardness distribution in the NiAl-AlMg6 joint interface.

Figure 13 shows the microhardness measurement results of AlMg6 and NiAl. The average microhardness of NiAl was 530 HV, and that of AlMg6 was 150 HV. The average microhardness of the initial AlMg6 was 165 HV. Therefore, after SHS, the microhardness of AlMg6, taking into account the measurement error, remained at the level of the initial values.

In addition, an experiment was conducted in which impurity gases were allowed to escape from the SHS reactor during synthesis by loosely tightening the top and bottom covers. The height of the Ti-C pellet was 20 mm.

**Figure 14:**
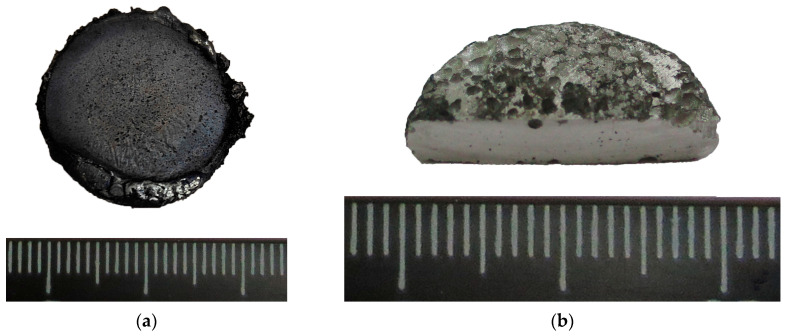
Surfaces after SHS: (**a**) NiAl with AlMg6; (**b**) AlMg6 with NiAl.

The experiment showed that the compound does not form when the SHS reactor is incompletely sealed (Figure 14). A burr formed around the perimeter of the synthesized NiAl caused by the molten NiAl, indicating that the temperature in the reactor was above 1640 °C (which corresponds to the melting point of NiAl). A large number of pores were found on the NiAl surface that was in contact with AlMg6. The AlMg6 layer had a small number of spherical pores. This indicates that there was no volumetric melting of AlMg6, and the melt did not flow into the cylindrical pores.

**Figure 15:**
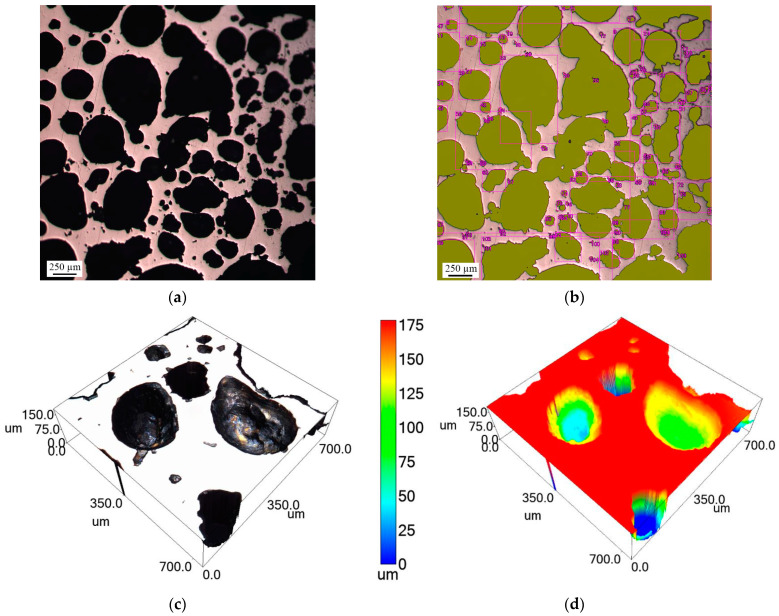
Microstructure of the NiAl surface (**a**); estimation of porosity (**b**); confocal 3D image (**c**); pore depth (**d**).

Figure 7a shows the porous structure of the NiAl surface that was in contact with the AlMg6 surface during the SHS process. The porosity of the sample was about 64% (Figure 15b). The average pore size was 188 µm in the horizontal direction and 176 µm in the vertical direction (Figure 15b). The pores have an elongated shape, directed into the depth of the sample (Figure 15c) from 90 to 155 µm (Figure 15d). Unfortunately, the confocal laser scanning microscope cannot view deeper pores and construct a 3D image.

**Figure 16:**
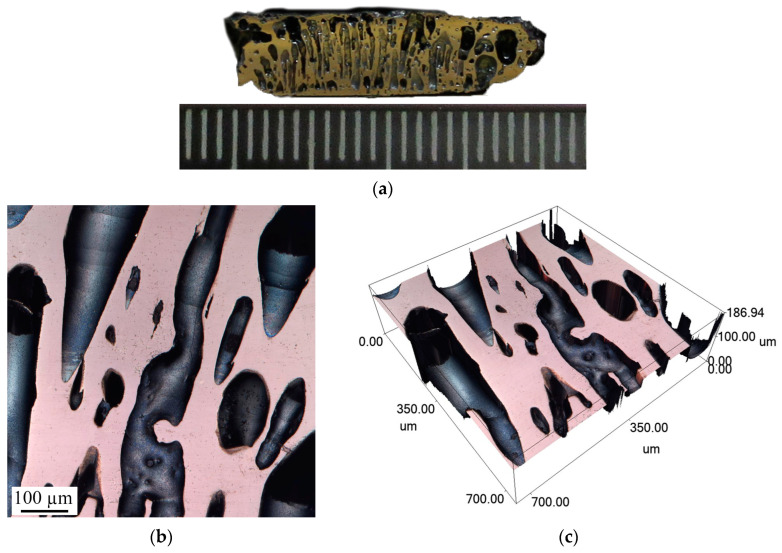
Structure of NiAl cross section (**a**); microstructure of central region (**b**); and confocal image (**c**) of NiAl through channels.

Microstructural studies of the central part of the NiAl sample revealed cylindrical elongated pores (Figure 16a) distributed throughout the volume of NiAl. This structure provides evidence for the high internal pressure of impurity gases in the reaction zone and indicates the direction of their flow during NiAl synthesis. The formed cylindrical pores inside NiAl have a continuous smooth surface (Figure 16b,c), which can positively influence the strength of the intermetallic compound [24].

## 4. Conclusions

1. The interaction of NiAl with AlMg6 during SHS in a sealed reactor occurred by a diffusion mechanism. Cylindrical pores in NiAl were filled with AlMg6 melt to a depth of up to 300 microns.
2. The compound cannot be formed when the SHS reactor is incompletely sealed or the pressure of impurity gases is low. The pressure of impurity gases produced during synthesis at the interface of NiAl and AlMg6 melt leads to the breaking of diffusion processes and the contact between the materials to be joined.
3. The mechanism of pore formation in AlMg6 during SHS was proposed. When the combustion front moves, hydrogen contained in PTS-1 accumulates in a narrow layer of liquid metal adjacent to the front and passes into the AlMg6 layer during crystallization. Spherical voids filled with hydrogen remain in the surface layer of AlMg6 due to the high crystallization rate of liquid metal, forming pores.
4. Elongated unidirectional pores uniformly distributed in the NiAl layer were formed during SHS, providing anisotropic properties of the material. The formation of lotus-type pores is associated with the passage of hydrogen and impurity gases through solidifying NiAl under the pressure of gaseous reaction products in a closed volume.
5. The measured total area of chaotically oriented pores in NiAl was about 30%, while the area of lotus-type pores was about 60%. This shows that the material with a different pore shape and porosity can be obtained by varying the height of the Ti-C pellet, which is the main source of impurity gases and hydrogen in the SHS process.
6. Thus, high-temperature composites with lotus-type pores can be produced by SHS in a closed reactor using the chemical reaction energy and the pressure of impurity gases and hydrogen without special equipment in one technological step.

## Data Availability

Data are contained within the article.
